# Mini-Arc for the Treatment of Female Stress Urinary Incontinence: Long-Term Prospective Evaluation by Patient Reported Outcomes

**DOI:** 10.1155/2014/659383

**Published:** 2014-01-20

**Authors:** Rui Oliveira, Alexandre Resende, Carlos Silva, Paulo Dinis, Francisco Cruz

**Affiliations:** ^1^Faculdade de Medicina da Universidade do Porto, Alameda Professor Hernâni Monteiro, 4200-319 Porto, Portugal; ^2^Serviço de Urologia, Centro Hospitalar São João, Alameda Professor Hernâni Monteiro, 4200-319 Porto, Portugal

## Abstract

Single-incision slings were introduced in the surgical treatment of female stress urinary incontinence (SUI) to lessen the morbidity associated with traditional midurethral slings. However, long-term reports on patient satisfaction are still scarce. This study describes the outcome of women treated with Mini-Arc at a mean follow-up of 45 months. In a previous report on 105 women with 15-month mean follow-up, 84 (80%) were found cured and 12 (11%) improved. Now, with a mean follow-up of 45 months, cured/improved patients were reassessed by telephone and completed Patient Global Impression of Improvement (PGI-I), Patient Global Impression of Severity (PGI-S), rated their improvement in a 0–100 scale, and answered if they would recommend the procedure. At 45-month follow-up, 73 women cured/improved were available for evaluation. Over 80% of the cured patients rated the improvement of SUI by the PGI-I as “very much better” or “much better,” reported their urinary tract condition to be “normal” on PGI-S, and described their improvement >70%. Ninety percent would recommend this procedure to a friend. The improved-patient population is very small (*n* = 7). This study shows that the majority of patients cured/improved after Mini-Arc placement maintain a high degree of satisfaction at a long-term evaluation.

## 1. Introduction

According to the European Association of Urology Guidelines on Urinary Incontinence, concerning the treatment of female stress urinary incontinence (SUI), the retropubic insertion of a midurethral synthetic sling (MUS) gives equivalent patient-reported cure of SUI at 12 months, when compared to colposuspension [1]. These guidelines also report that midurethral synthetic sling inserted by either the transobturator (TO) or retropubic (RP) route gives equivalent patient-reported outcome at 12 months [1].

With an obvious trending towards less and less invasive surgical options, single-incision vaginal slings (SIS) have emerged. They require very limited intracorporeal dissection, proposing to further increase safety of suburethral slings, without jeopardizing the success rates reported by conventional RP and TO access [2]. These SIS outcomes are comparable with conventional MUS at short-term follow-up [3–5]. Although sparse, two-year follow-up studies are available and seem to maintain steady success rates over this time [6, 7]. Longer follow-up time reports are needed, to ensure that, in the long run, these SIS offer constant success rates.

The objective of this study is to describe the outcome of women treated with Mini-Arc at a mean follow-up of 45 months, based on a baseline population which has already been reported in a short-term paper [8], after adequate long-term follow-up evaluation. Previously considered cured and improved patients were evaluated to access if their condition remains stable, as reflected in a subjective satisfaction evaluation.

## 2. Materials and Methods

This is a single-centre prospective evaluation of women with urodynamic stress urinary incontinence, which were submitted to Mini-Arc (American Medical Systems, Minnetonka, MN, USA) placement as a primary surgical treatment. Surgical technique, inclusion and exclusion criteria, baseline population characteristics, and short-term outcome and complications have already been described in a previous paper [8]. On this report, on 105 women with a mean follow-up of 15 months (and a minimum follow-up of 6 months), 84 patients (80%) were found cured and 12 (11%) improved. Now, with a mean follow-up of 45 months, cured/improved patients were reassessed by telephone interview and completed Patient Global Impression of Improvement (PGI-I), to access treatment response [9], Patient Global Impression of Severity (PGI-S), to access current SUI condition [9], rated their improvement in a 0–100 scale, and answered if they would recommend the procedure. This study was approved by the institutions' ethics committees and each participant provided written informed consent prior to enrollment.

## 3. Results and Discussion

**Figure 1:**
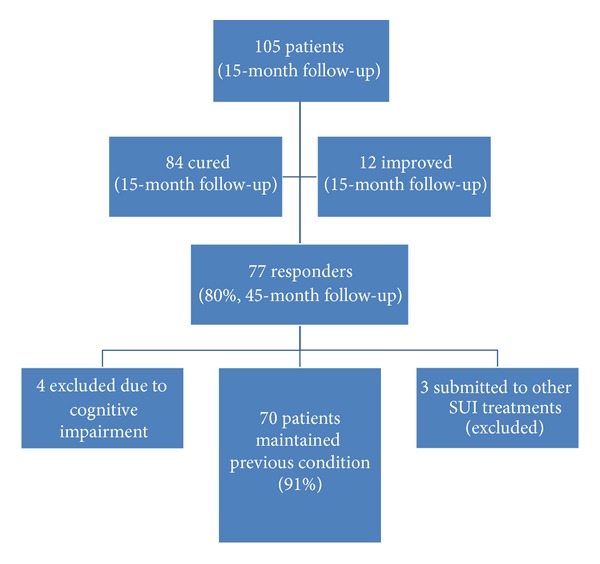
Study subject distribution tree.

At 15-month mean follow-up (initial population of 105 patients), 84 patients were cured and 12 improved. Seventy-seven patients could be contacted (80% of the initial population) and have a current mean follow-up of 45 months (median 43.5 months). Four had to be excluded due to cognitive impairment. Three were submitted to other forms of SUI treatment during the period of follow-up. So, from a total of 77 responders, 70 (91%) maintained the initial cure/improvement situation (Figure 1). Subsequently, 63 previously considered cured and 7 improved were available for analysis.

**Figure 2:**
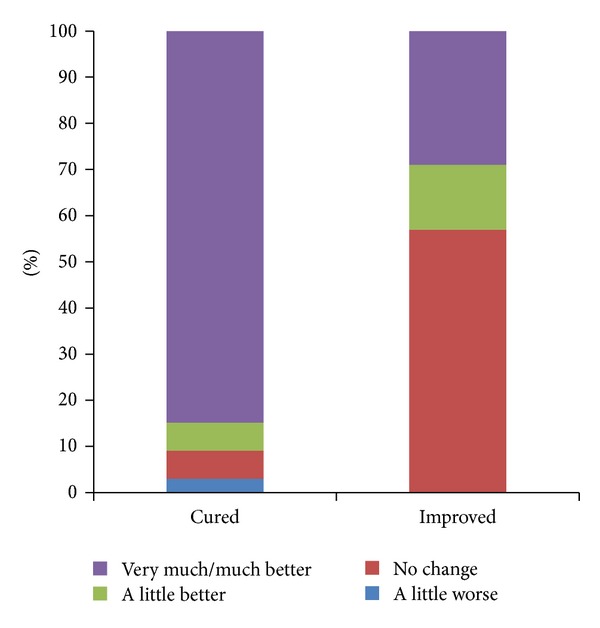
Patient Global Impression of Improvement (PGI-I).

**Figure 3:**
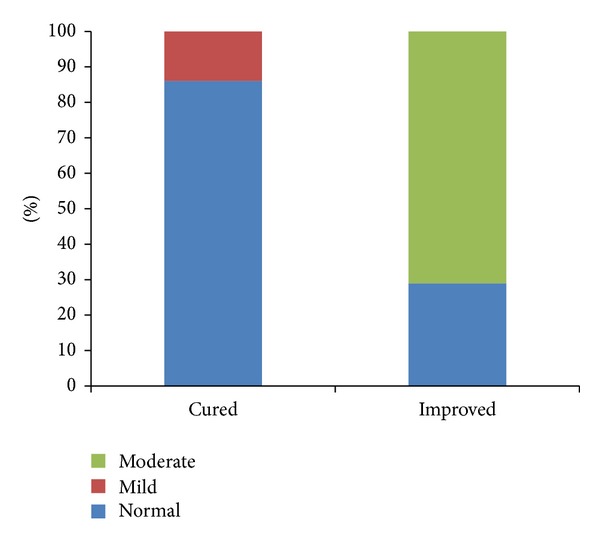
Patient Global Impression of Severity (PGI-S).

Fifty-three of the cured patients (84%) rated the improvement of SUI by the PGI-I as “very much better” or “much better” and 4 (6%) considered it to be “a little better.” Four patients (6%) answered “no change” and two (3%) “a little worse” (Figure 2). The mean rate of improvement in a 0–100 scale was 81 ± 15, 52 patients (83%) rating improvement >70. Fifty-four patients (86%) reported their urinary tract condition (UTC) to be “normal” on PGI-S (Figure 3). Fifty-seven (90%) would recommend this procedure to a friend.

When analyzing improved patients (*n* = 7), 2 (29%) considered their PGI-I as “very much better” or “much better,” 1 (14%) “a little better,” and 4 (57%) “no change” (Figure 2). Only 3 patients (43%) rated their improvement to be equal or superior to 70% in a 0–100 score or would recommend the procedure to a friend. Five patients (71%) answered “moderate” on PGI-S, with only two patients (29%) considering their UTC to be “normal” (Figure 3).

Female urinary incontinence is a very common condition, which can affect around 35% of women; SUI is the most prevalent type, but the consultation and treatment rates are very low [10].

The conservative management is the first treatment option and it usually includes pelvic floor muscle training, which can be very successful in around a fourth of the patients, especially in younger patients with mild forms of the condition [11]. Obese women can adopt a program of weight reduction associated with physical exercise, which can offer a 25% cure rate, since they stay firmly devoted to the program over time and are willing to wait for the improvements [12]. As a result, surgery is the most common form of SUI treatment worldwide. During the last 2 decades we have observed the development of promising SUI surgical techniques and the introduction of suburethral, tension-free slings. TVT (Gynecare, Ethicon, Somerville, New Jersey, USA) was the first device of this kind to be introduced in clinical practice, in 1996 by Ulmsten et al. [13].

According to the European Association of Urology Guidelines on Urinary Incontinence, the RP insertion of a MUS gives equivalent patient-reported cure of SUI at 12 months, when compared to colposuspension [1]. Nonetheless, TVT shows low invasiveness, short hospital stay, reduced risk of prolonged catheterization, and low risk of causing future pelvic organ prolapsed [14]. All together, these characteristics were responsible for the swift replacement of Burch colposuspension as the preferred surgical approach to female SUI [14]. TVT has become the gold standard in the surgical treatment of SUI with high cure rates that subsist at long time follow-up [15]. The blind passage of needles through the RP space was associated with severe complications, such as bladder and bowel perforations and life-threatening vascular injuries [16, 17]. These concerns led to the development of the TO route in 2000, a relatively avascular space for the passage of trocars [18]. However, TO tapes have been associated with prolonged and limitative pain referred to the groin and upper thigh, due to the obturator foramen violation and vaginal perforations due to a more horizontal trajectory of the needle passage [16, 17, 19].

To our knowledge, this is the longest follow-up prospective report on Mini-Arc single-incision sling. At roughly four-year follow-up, the majority of patients cured or improved at short-term evaluation maintain a high degree of satisfaction at long term.

Short- and midterm reports on Mini-Arc have, on the majority, been consistent with the initial results of this series [6, 7] and comparable to conventional MUS [20, 21], with a low morbidity profile [20, 21].

The number of patients available for this evaluation, with 80% responders at almost 4-year mean follow-up, permits having an adequate idea of the long-term outcomes of the initial population, in a reliable way.

Patient Global Impression of Improvement questionnaire addresses the SUI treatment outcomes when compared with baseline condition and the results among the cured patients describe a 90% (57 patients) positive result, with 84% of the patients considering their actual condition to be “very much better” or “much better,” which is usually assumed to be equal to a cured situation. These numbers are certainly reliable, as the 0–100 improvement scale results mean score is over 80%, with over than 4/5 of the cured patients rating this improvement >70%. On the other way, the actual urinary tract condition, addressed by PGI-S, is considered “normal” by 86% of the cured women. Only 10% of the cured women did not recommend the procedure to a friend.

The improved-patient population is very small (*n* = 7), and interpreting their results would not prompt solid conclusions.

These reports on long-term evaluation are very important to assure that SIS are a valid technique, with fair and comparable results at short- and middle-term evaluations, and that over time the results are maintained stable.

## 4. Conclusions

This study shows that the majority of patients cured/improved after Mini-Arc placement maintain a high degree of satisfaction at a long-term evaluation.
